# Supplementary figures and images for: The interplay of RNA:DNA hybrid structure and G-quadruplexes determines the outcome of R-loop-replisome collisions
Source: eLife. 2021 Sep 8;10:e72286. doi: 10.7554/eLife.72286 (PMC8479836; doi:10.7554/eLife.72286)

Figure 1 - source data 2

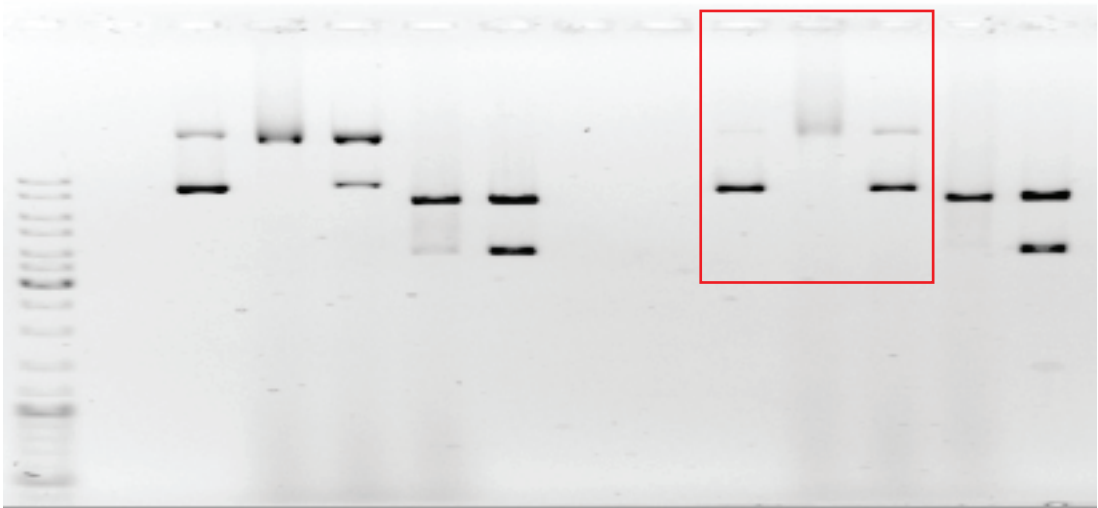

Figure 1D

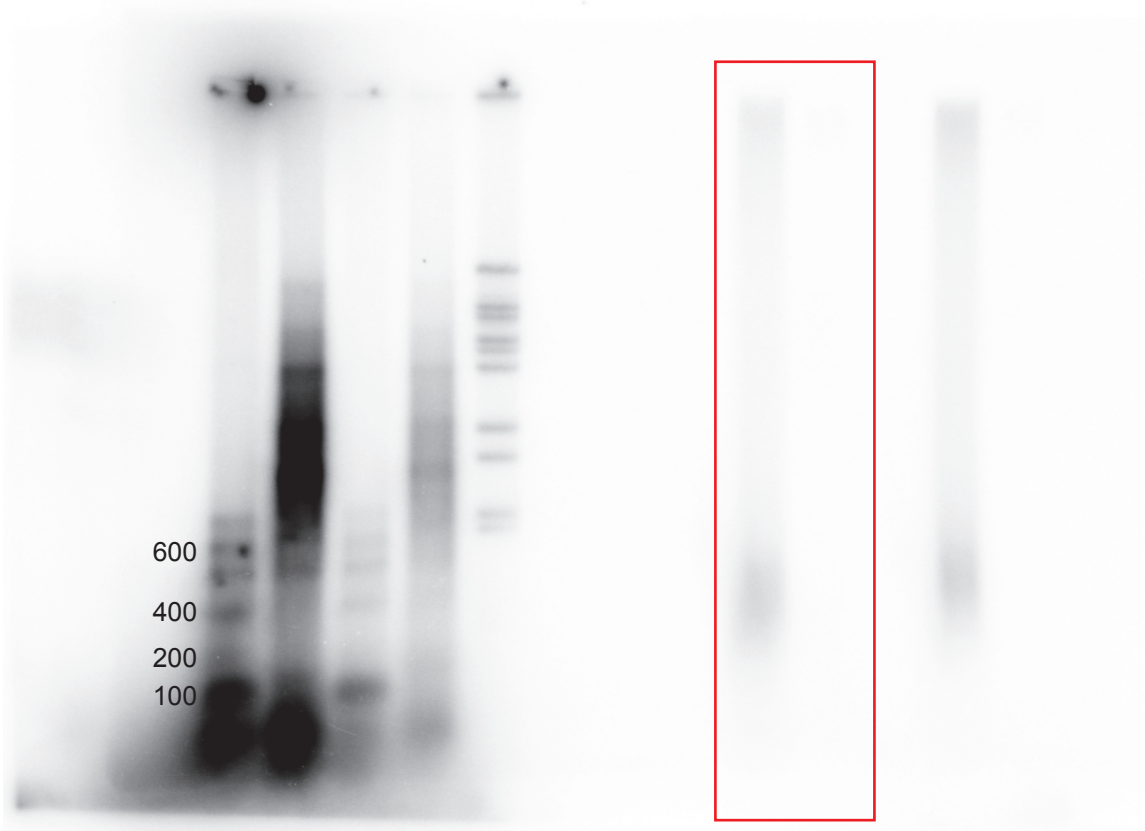

Figure 1E

Supplement: Figure 1—source data 2. [file elife-72286-fig1-data2.pdf]

Figure 1 - source data 3

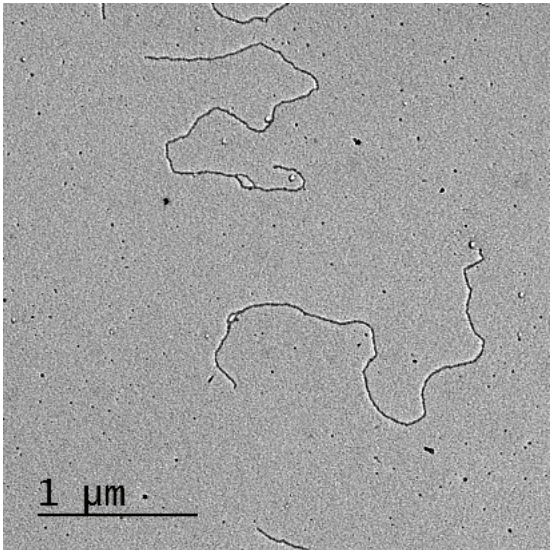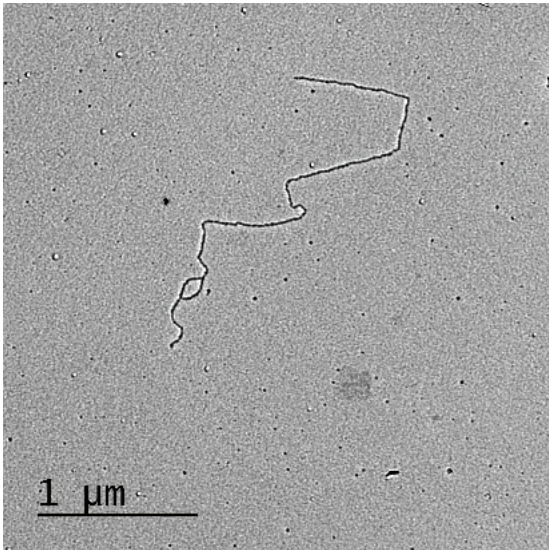

T4 gp32

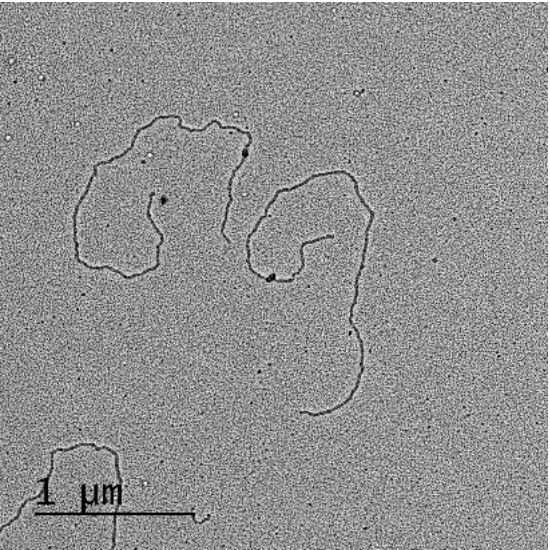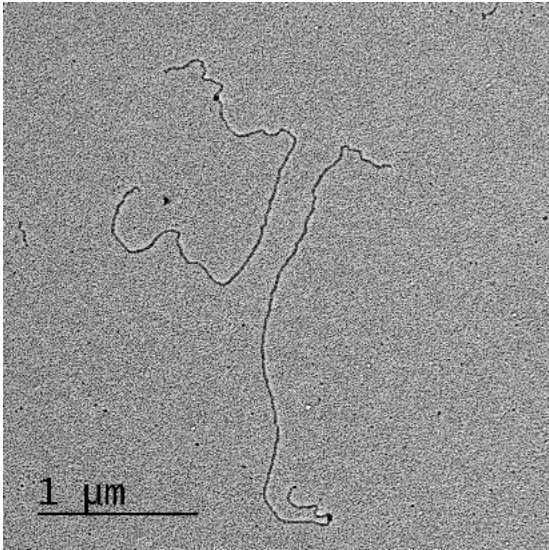

S9.6

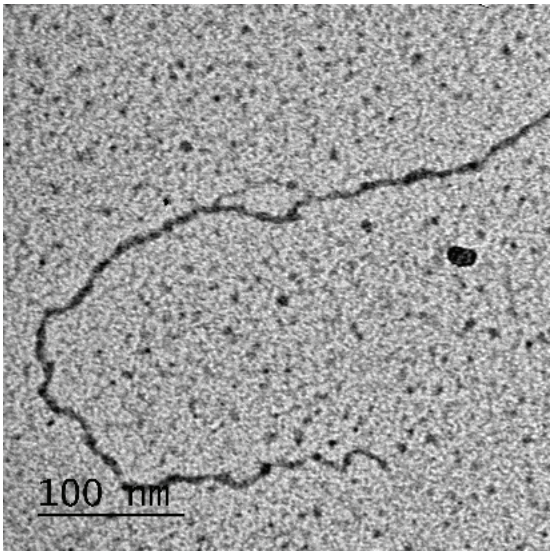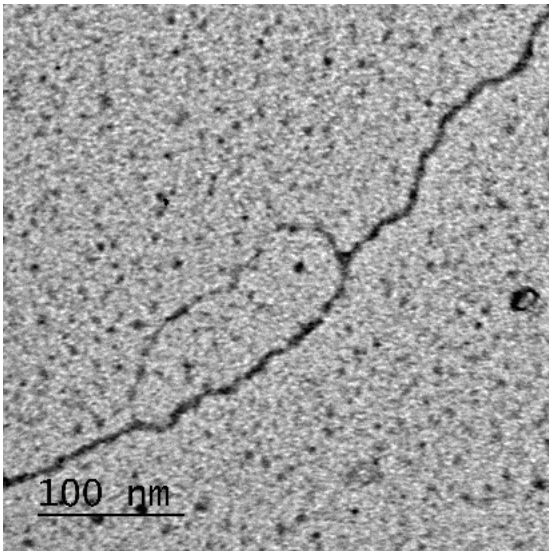

Formamide

Figure 1F

Supplement: Figure 1—source data 3. [file elife-72286-fig1-data3.pdf]

Figure 1- figure supplement 1 - source data 1

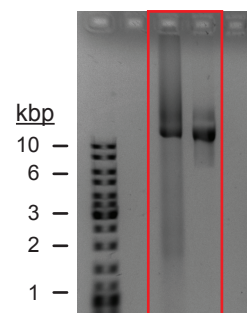

Figure 1 - figure supplement 1B

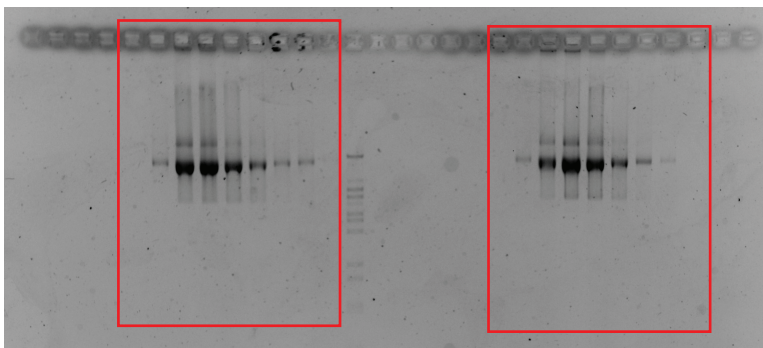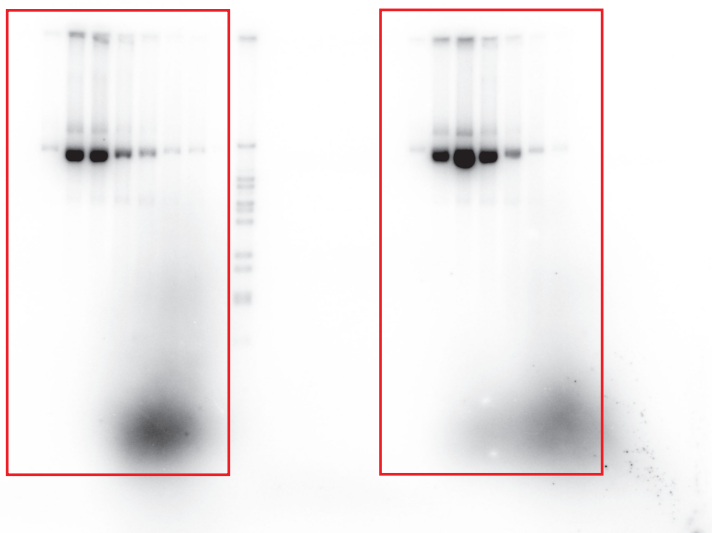

Figure 1 - figure supplement 1C

Supplement: Figure 1—figure supplement 1—source data 1. [file elife-72286-fig1-figsupp1-data1.pdf]

Figure 1- figure supplement 1 - source data 2

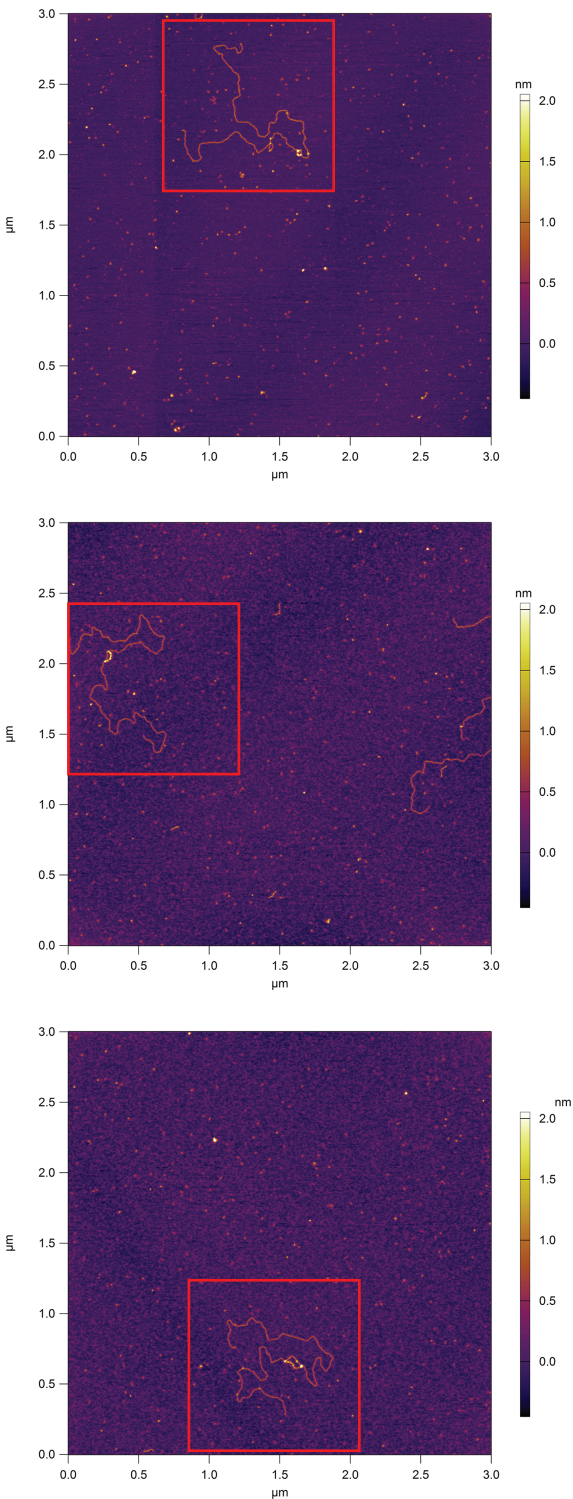

Figure 1 - figure supplement 1D

Supplement: Figure 1—figure supplement 1—source data 2. [file elife-72286-fig1-figsupp1-data2.pdf]

## Figure 2 - source data 1

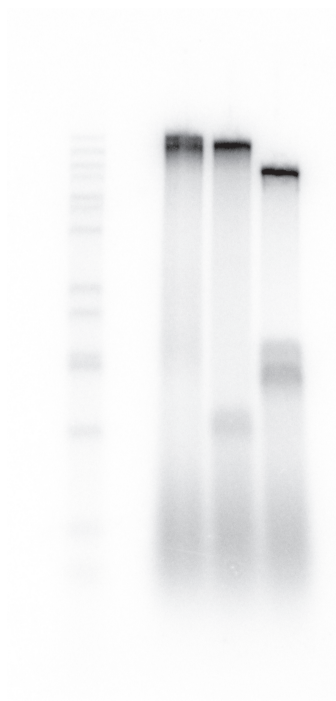

Figure 2B

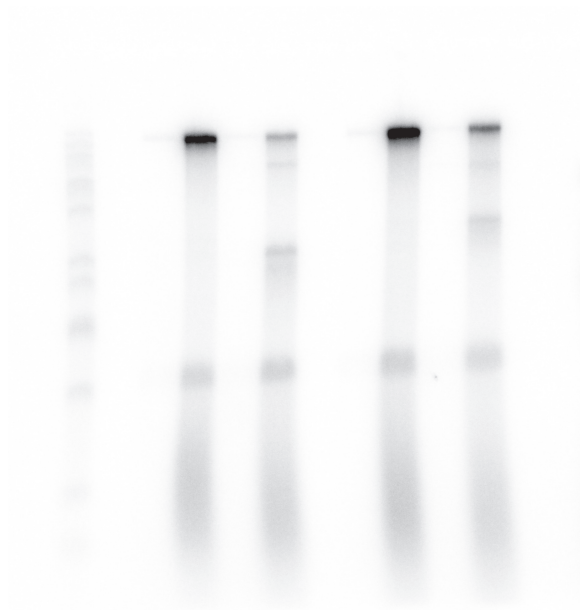

Figure 2C - denaturing

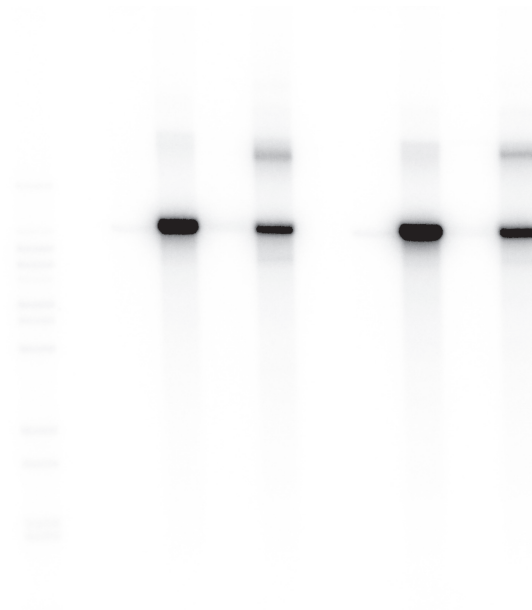

Figure 2C - native

Supplement: Figure 2—source data 1. [file elife-72286-fig2-data1.pdf]

Figure 2 - source data 2

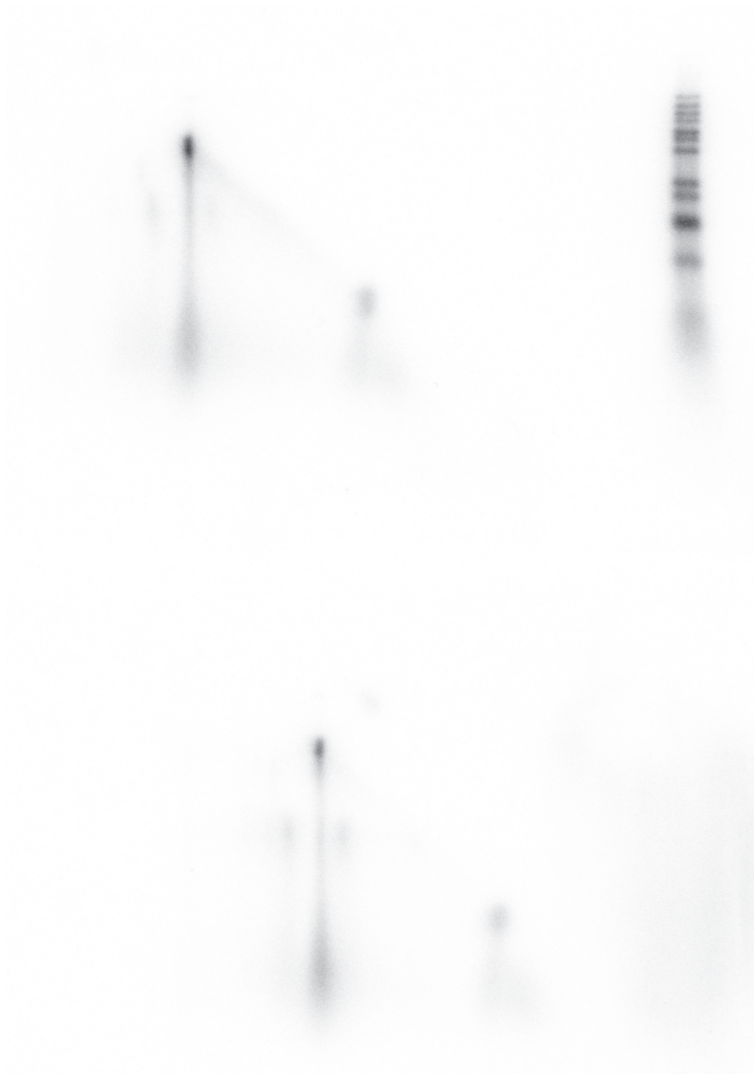

Figure 2E - 2nd dimension (alkaline)

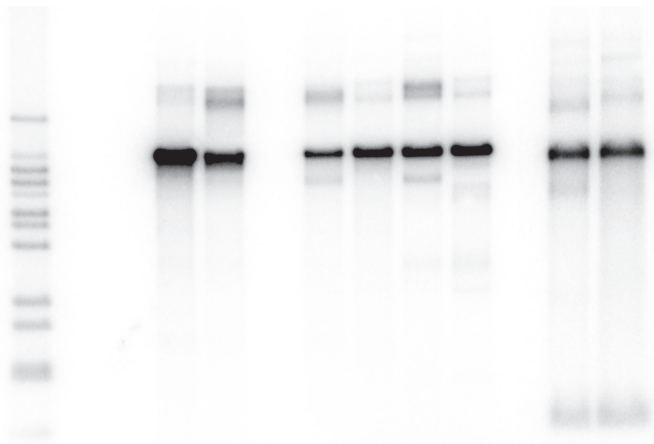

Figure 2E - 1st dimension (native)

Supplement: Figure 2—source data 2. [file elife-72286-fig2-data2.pdf]

Figure 2 - source data 3

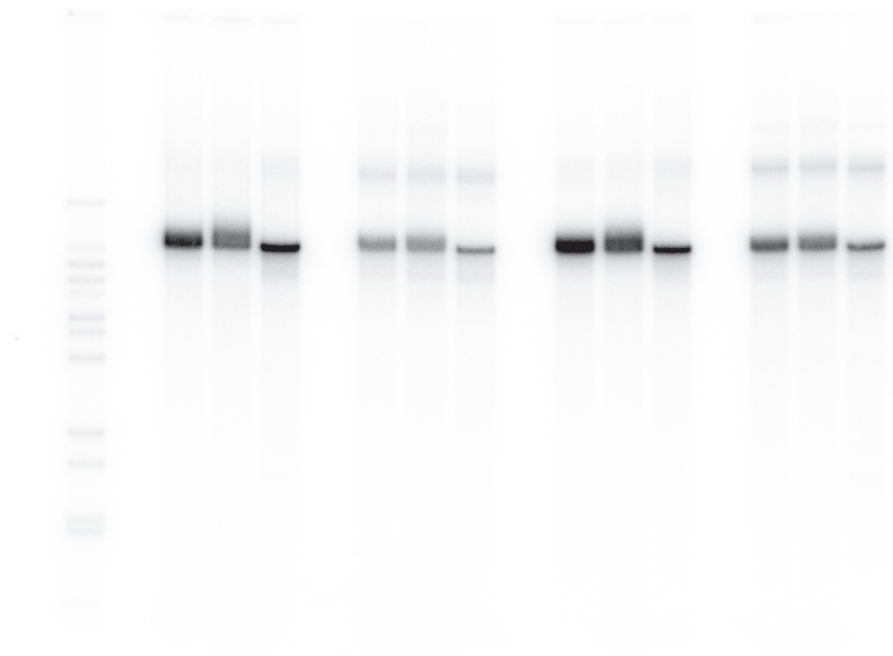

Figure 2F - native

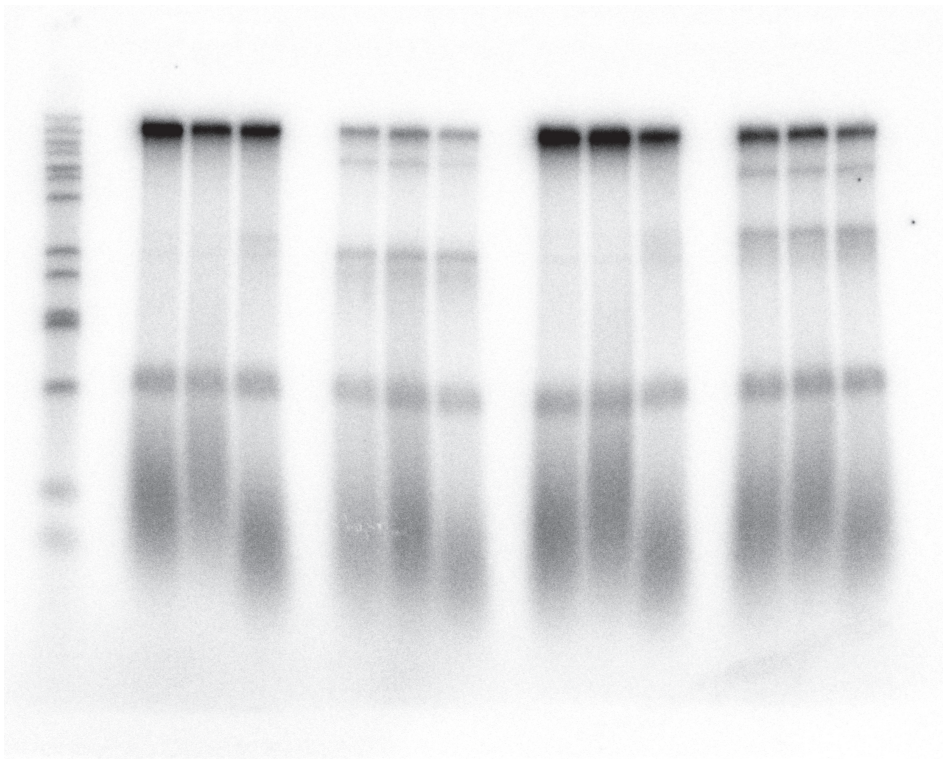

Figure 2F - denaturing

Supplement: Figure 2—source data 3. [file elife-72286-fig2-data3.pdf]

**Figure 5 - source data 1**

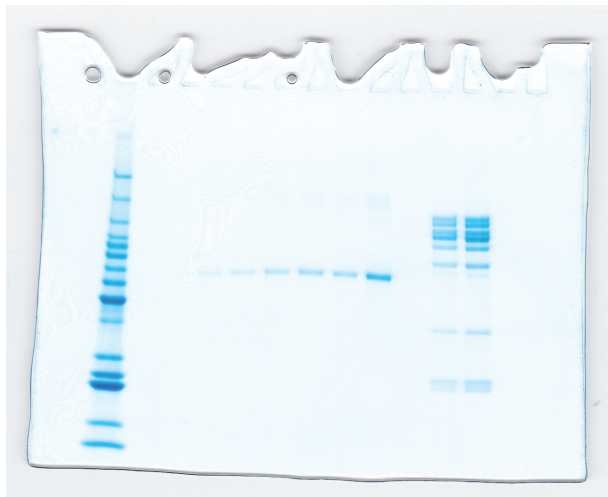

Figure 5A

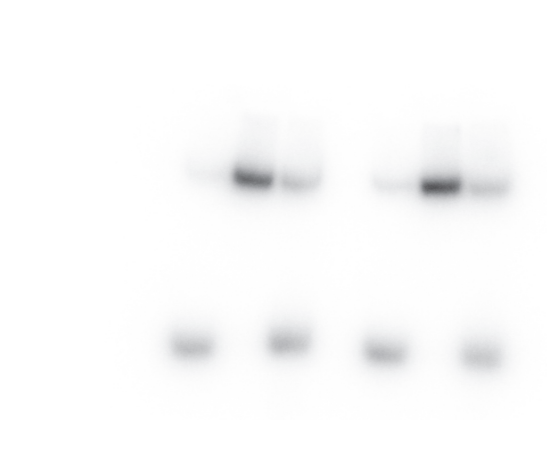

Figure 5B, i

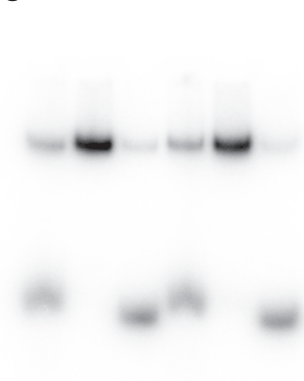

Figure 5B, ii

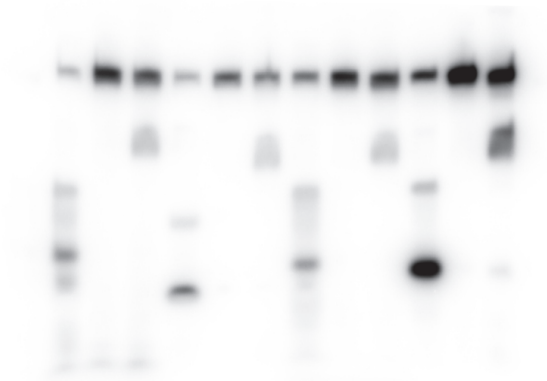

Figure 5B, iii+iv

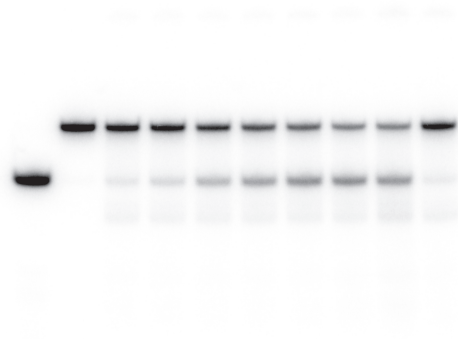

Figure 5C, DNA:DNA

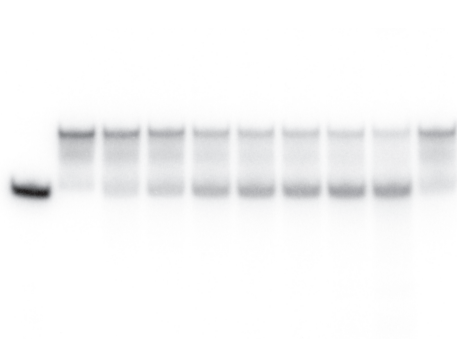

Figure 5C, RNA:DNA

Supplement: Figure 5—source data 1. [file elife-72286-fig5-data1.pdf]

**Figure 5 - source data 2**

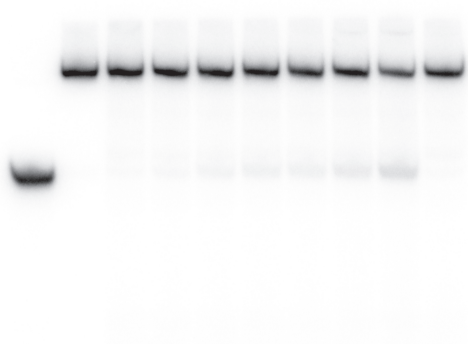

Figure 5D, G4-wt

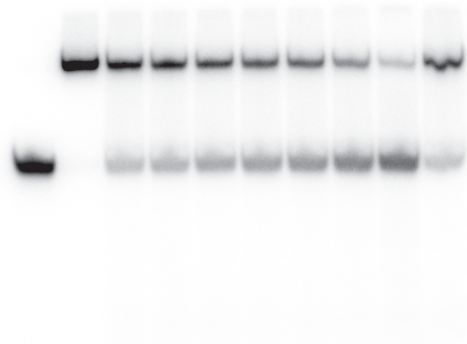

Figure 5D, G4-mut

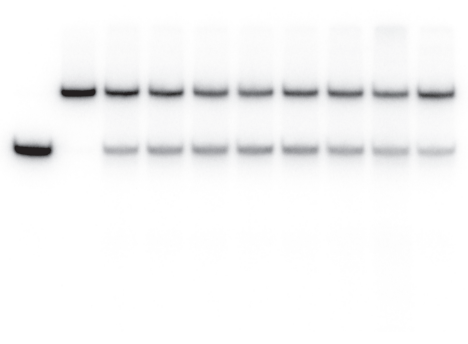

Figure 5E, G4-wt

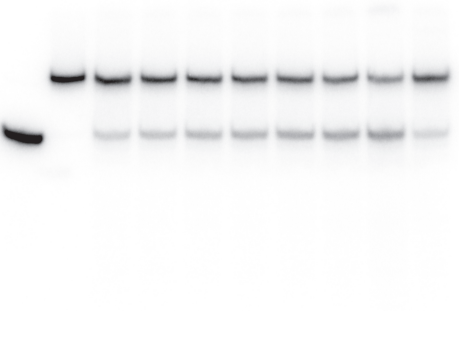

Figure 5E, G4-mut

Supplement: Figure 5—source data 2. [file elife-72286-fig5-data2.pdf]

**Figure 7 - figure supplement 1 - source data 1**

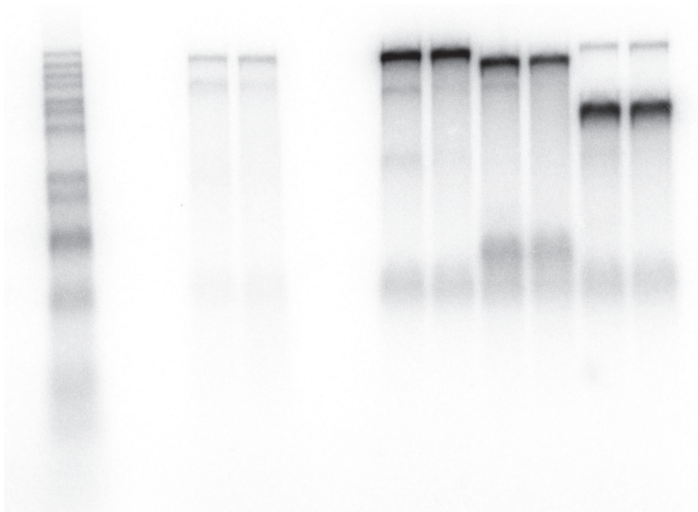

Figure 7 - figure supplement 1A

Supplement: Figure 7—figure supplement 1—source data 1. [file elife-72286-fig7-figsupp1-data1.pdf]
