# Supplementary material for: The interplay of RNA:DNA hybrid structure and G-quadruplexes determines the outcome of R-loop-replisome collisions
Source: eLife. 2021 Sep 8;10:e72286. doi: 10.7554/eLife.72286 (PMC8479836; doi:10.7554/eLife.72286)
Supplement: Figure 2—figure supplement 1—source data 2. [file elife-72286-fig2-figsupp1-data2.pdf]

Figure 2 - figure supplement 1 - source data 2

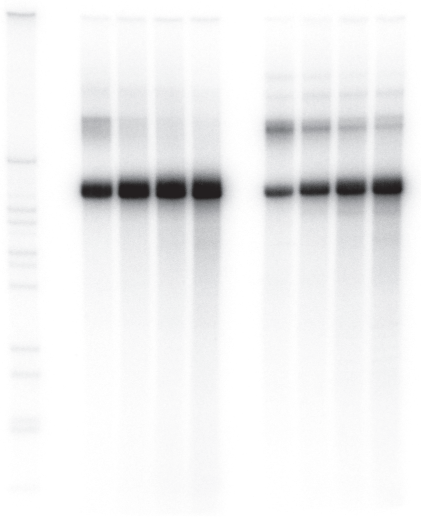

Figure 2 - figure supplement 1B - HO, native

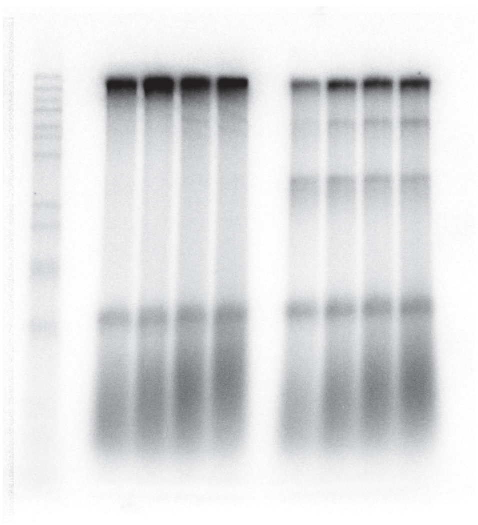

Figure 2 - figure supplement 1B - HO, denaturing
