# Supplementary material for: The interplay of RNA:DNA hybrid structure and G-quadruplexes determines the outcome of R-loop-replisome collisions
Source: eLife. 2021 Sep 8;10:e72286. doi: 10.7554/eLife.72286 (PMC8479836; doi:10.7554/eLife.72286)
Supplement: Figure 3—source data 1. [file elife-72286-fig3-data1.pdf]

Figure 3 - source data 1

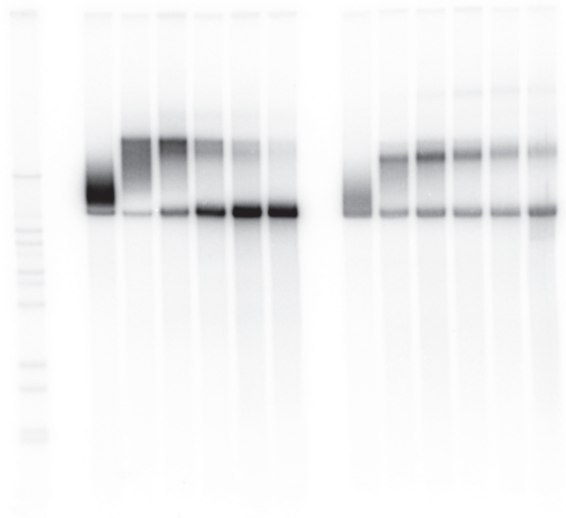

Figure 3 -
CD, native

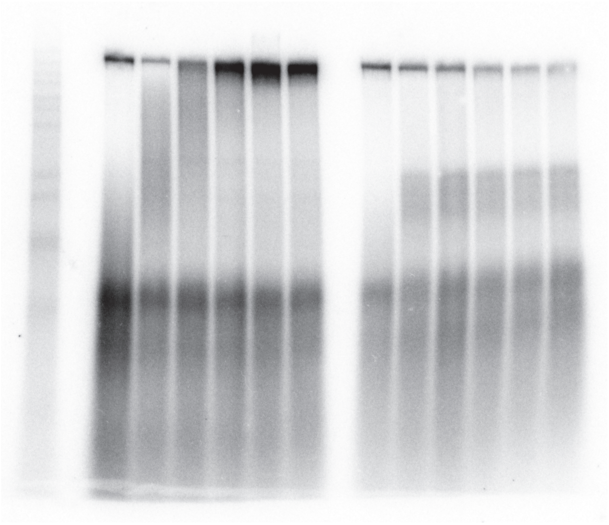

Figure 3 -
CD, denaturing

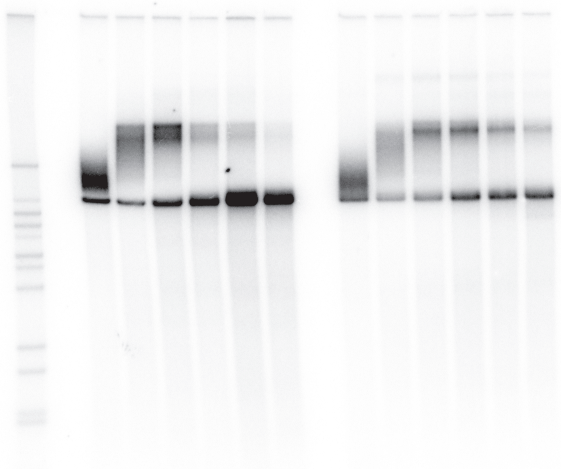

Figure 3 -
HO, native

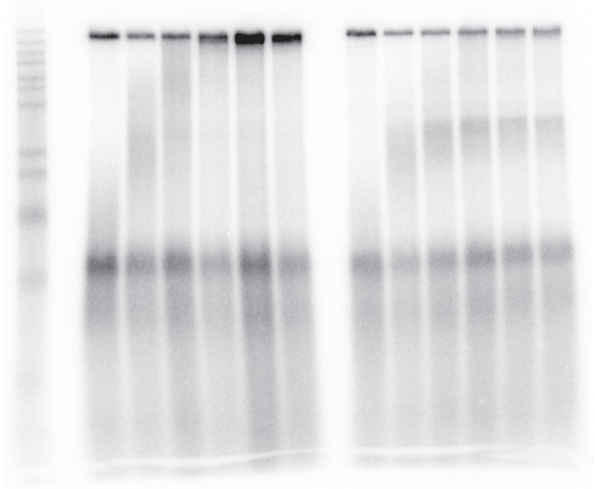

Figure 3 -
HO, denaturing
