# Supplementary material for: The interplay of RNA:DNA hybrid structure and G-quadruplexes determines the outcome of R-loop-replisome collisions
Source: eLife. 2021 Sep 8;10:e72286. doi: 10.7554/eLife.72286 (PMC8479836; doi:10.7554/eLife.72286)
Supplement: Figure 4—source data 2. [file elife-72286-fig4-data2.pdf]

Figure 4 - source data 2

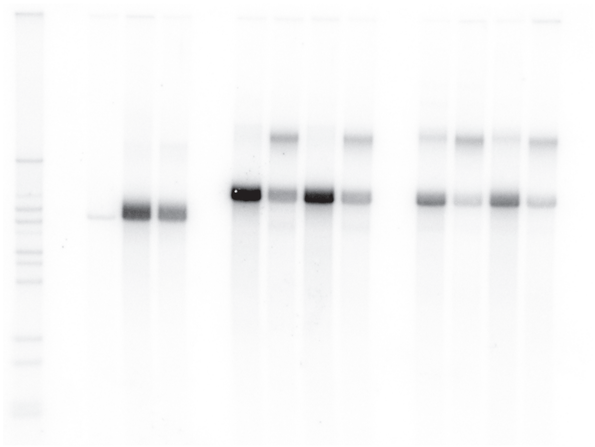

Figure 4D - native

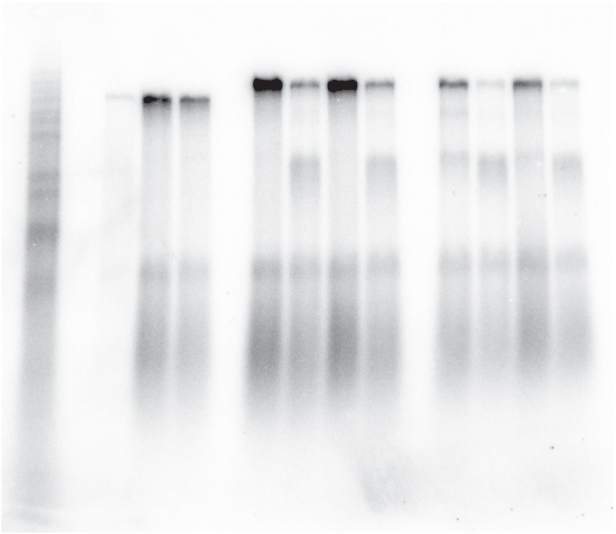

Figure 4D - denaturing

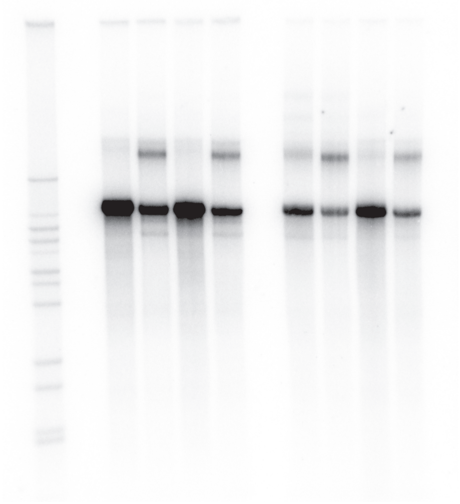

Figure 4E - lanes 1-8 - native

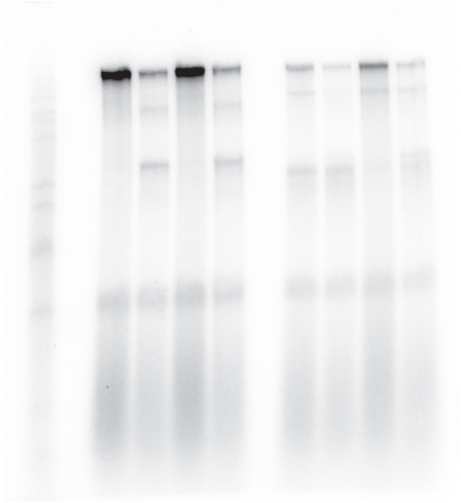

Figure 4E - lanes 1-8- denaturing

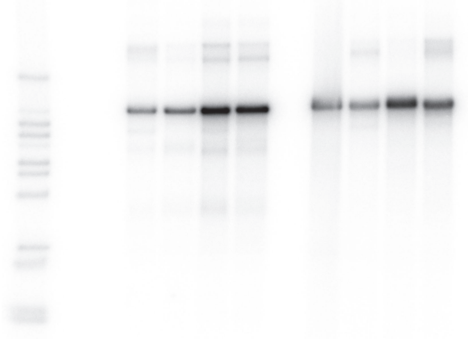

Figure 4E - lanes 9-12- native

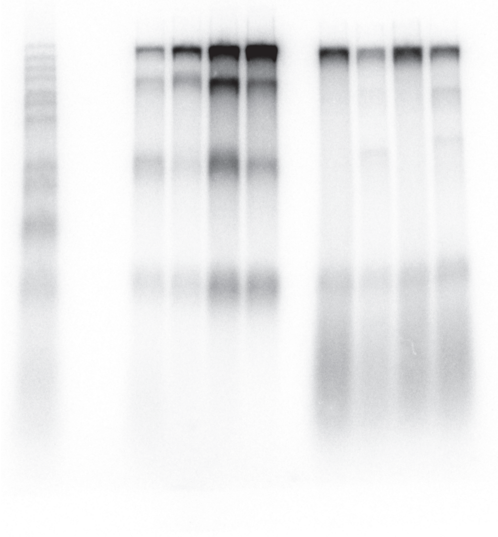

Figure 4E - lanes 9-12- denaturing
